# Equity in public health standards: a qualitative document analysis of policies from two Canadian provinces

**DOI:** 10.1186/1475-9276-11-28

**Published:** 2012-05-25

**Authors:** Andrew D Pinto, Heather Manson, Bernadette Pauly, Joanne Thanos, Amanda Parks, Amy Cox

**Affiliations:** 1 Department of Family and Community Medicine, St. Michael’s Hospital, 410 Sherbourne Street, 4th Floor, Toronto, Ontario, Canada, M4X 1K2; 2 Centre for Research on Inner City Health, Keenan Research Centre, Li Ka Shing Knowledge Institute, St. Michael’s Hospital, 209 Victoria Street, 3rd floor, Toronto, Canada, M5B 1T8; 3 Public Health Ontario, 480 University Avenue, Suite 300, Toronto, Ontario, Canada, M5G 1V2; 4 School of Nursing, University of Victoria, Box 1700 STN CSC, Victoria, British Columbia, Canada, V8W 2Y2; 5 Centre For Addictions Research of British Columbia, 273-2300 McKenzie Avenue, Victoria, British Columbia, Canada, V8P 5C2; 6 Public Health Division, Ministry of Health and Long-Term Care, 393 University Avenue, 21st Floor, Toronto, Ontario, Canada, M7A 2S1; 7 Interior Health Authority, 220-1815 Kirschner Road, Kelowna, British Columbia, Canada, V1Y 4N7

**Keywords:** Public health, Social justice, Health equity, Health planning, Standards

## Abstract

**Introduction:**

Promoting health equity is a key goal of many public health systems. However, little is known about how equity is conceptualized in such systems, particularly as standards of public health practice are established. As part of a larger study examining the renewal of public health in two Canadian provinces, Ontario and British Columbia (BC), we undertook an analysis of relevant public health documents related to equity. The aim of this paper is to discuss how equity is considered within documents that outline standards for public health.

**Methods:**

A research team consisting of policymakers and academics identified key documents related to the public health renewal process in each province. The documents were analyzed using constant comparative analysis to identify key themes related to the conceptualization and integration of health equity as part of public health renewal in Ontario and BC. Documents were coded inductively with higher levels of abstraction achieved through multiple readings. Sets of questions were developed to guide the analysis throughout the process.

**Results:**

In both sets of provincial documents health inequities were defined in a similar fashion, as the consequence of unfair or unjust structural conditions. Reducing health inequities was an explicit goal of the public health renewal process. In Ontario, addressing “priority populations” was used as a proxy term for health equity and the focus was on existing programs. In BC, the incorporation of an equity lens enhanced the identification of health inequities, with a particular emphasis on the social determinants of health. In both, priority was given to reducing barriers to public health services and to forming partnerships with other sectors to reduce health inequities. Limits to the accountability of public health to reduce health inequities were identified in both provinces.

**Conclusion:**

This study contributes to understanding how health equity is conceptualized and incorporated into standards for local public health. As reflected in their policies, both provinces have embraced the importance of reducing health inequities. Both concepualized this process as rooted in structural injustices and the social determinants of health. Differences in the conceptualization of health equity likely reflect contextual influences on the public health renewal processes in each jurisdiction.

## Introduction

In Canada, public health is the shared responsibility of governments at the municipal, provincial/territorial and federal level [1]. Over the past decade, a number of major reports have called for a renewal of the national public health system [2-4]. Specific plans have included calls for increased collaboration between all levels of government, the integration of surveillance systems across the country and the delineation of clearer lines of authority. The dual goals of public health renewal in Canada are to improve the overall health of the population and to reduce health inequities. Health inequities are differences in health which are unnecessary, avoidable and considered unfair and unjust [2,5].

A key mechanism of renewal is the development of standards to guide local public health practice [6]. Standards are seen as a way to improve accountability, improve equity in access, assist in measuring the impact of services and to secure resources for public health programs [7-10]. The process of creating and implementing standards has shaped the mandate and clarified and broadened the scope of public health services [11,12].

In Canada, the ten provincial and three territorial governments have constitutional responsibility for health care and provide the majority of the financial support to deliver these services at no cost to individuals. They set the direction of local public health programs and determine funding levels. The development of standards in each provincial and territorial jurisdiction has been influenced by different contextual factors, including public health legislation, history, leadership, goals and mandates. In this paper, we examine how equity is conceptualized and incorporated into the standards of two of Canada’s most populous provinces, Ontario and British Columbia (BC). This analysis is part of a broader program of research, the Renewal of Public Health Systems (RePHS) study. RePHS focuses on these two jurisdictions because they have experienced parallel renewal processes over the past decade.

As part of the overall study, we wanted to gain insight into how and to what extent an equity lens is incorporated into public health standards. Such a lens focuses attention on the differences in social conditions that shape health as a consequence of social positioning [13], and can be understood as “a metaphorical pair of glasses that ensures people ask ‘who will benefit?’” [14]. We aim to answer the question, how is equity conceptualized and integrated into key public health documents in Ontario and BC? Our findings provide a deeper understanding of the role of local public health in addressing health inequities and have the potential to enhance the incorporation of equity considerations into programs and services.

## Background

In Ontario, the *Mandatory Health Programs and Services Guidelines (MHPSG)* were developed in 1988 and revised in 1997. They explicitly defined the minimum requirements for services provided by local public health units [15]. Between 2006 and 2008, the MHPSG were reviewed as part of an overall strategy to improve public health capacity in Ontario [16]. An advisory committee guided the process, composed of key technical experts, practitioners and staff from the Ministries of Health and Long-Term Care, Children and Youth Services, and Health Promotion (now, Health Promotion and Sport). The *Ontario Public Health Standards (OPHS), 2008* established “requirements for fundamental public health programs and services, which include assessment and surveillance, health promotion and policy development, disease and injury prevention, and health” [17] (p. 1). Built on the principles of need, impact, capacity and partnership, and collaboration, the OPHS include one Foundational Standard and 13 Program Standards. These are grouped under the areas of communicable disease control, environmental health, emergency preparedness, chronic disease, injury prevention and family health. The Minister of Health issues these standards and the accountability for implementation rests with local Boards of Health governed at the municipal level. The development of the OPHS was influenced by four key factors [15,18]. First, the new standards were required to be revenue-neutral, hence a significantly expanded scope for public health could not be recommended. Second, there was an emphasis on looking at the scientific evidence to establish each standard. Third, a logic model approach was used to establish short- and long-term outcomes and develop a performance management system to better link actions with board and societal outcomes. Fourth, the OPHS were designed to be less prescriptive than the preceding MHSPG, in order to enable public health units to tailor their programs to meet local needs.

In BC, the development of the *Framework for Core Functions in Public Health, 2005* (BC Core Functions Framework) was part of a larger effort to renew public health in the province. No previous standards existed; hence there was a greater ability to define their scope than in Ontario. According to the BC Ministry of Health Services, “an effective public health system needs clearly defined essential functions. The BC Framework establishes these essential functions.” [19] (p. 15). The Population Health and Wellness Division of the Ministry of Health Services led the development of the BC Core Functions Framework, the related evidence and best practice reviews and the Model Core Program (MCP's) papers. The latter were developed jointly with the province’s six health authorities. The Framework identified the 21 core public health programs that health authorities are expected to provide, in the areas of health improvement, disease, injury and disability prevention, environmental health and health emergency management.

Unlike the OPHS, the MCP papers are considered a set of best practice documents, rather than standards that are mandated or enforced by legislation. As such, health authorities were free to determine their own priorities for a program, their own areas of focus for performance improvement and their own performance improvement targets. Their only obligation was to develop a plan that included specific elements (a base case, gap analysis, priority areas, performance improvement targets and performance improvement strategies) and report publicly.

Reducing health inequities has been identified as a key objective of public health renewal in both Ontario and British Columbia. The scope of the OPHS includes, “to promote the health of the population as a whole, and with community partners to reduce health inequities” [17] (p. 1). The BC Core Functions Framework lists the essential functions of public health as improving the overall health of the population, preventing disease and “to reduce inequalities in health” [19] (p. 12). The Core Functions Framework also explicitly includes both population and equity lenses to be used when implementing programs [20]. Given this background to public health renewal in each province, we analysed how health equity is conceptualized and integrated into each set of key documents.

## Methods

We conducted an inductive content analysis of the OPHS and Protocols and the BC Core Functions Framework available as of May 31, 2010 (Appendix). The study team consisted of both academic researchers and public health knowledge user partners. The latter contributed key insights into the interpretation of the data based on their experiences. In order to remain open to various conceptions of equity within the documents, all team members shared their own views on equity and how this influenced their reading of the documents. Further, the team guided and provided feedback on the analysis, which was carried out by one author (ADP). We employed constant comparative analysis drawing on grounded theory methodology. Constant comparative analysis is a useful approach to discover dominant social and structural processes to assist in explaining behaviour in certain situations [21]. The process of constant comparative analysis is emergent in that the findings are the outcome of multiple readings and subsequently higher levels of abstraction.

Throughout this process, memos were kept to capture ideas and questions about equity as understandings emerged and changed. These memos were developed at each stage of the review process, described below. The memos also served as a place to capture feedback on the analysis as it developed and to increase confidence in the themes that emerged. For each set of documents, the initial overview document was analysed, followed by the Protocols or MCP papers. All documents were entered into NVivo^TM^ 8.0 (QSR International, Cambridge, MA), which was used to code documents and organize notes.

To reach higher levels of abstraction, the documents were read, reread and coded in three stages. The questions developed by the study team are listed in Table 1. The first stage began with four preliminary questions. These addressed whether the terms “equity” or “inequity” were mentioned, including the context in which they were mentioned, definitions of health inequity, and whether reducing health inequities was an explicit goal. The answers to these questions generated a set of seven secondary questions, which focused the second reading and coding of the documents. The second reading focused on proxy terms used to discuss equity considerations, equity in relation to social justice, the social determinants of health (SDOH) and structural conditions that produce inequity, naming of specific populations as priorities, specific actions recommended and the extent to which accountability is considered. Finally, the answers to these questions generated a third set of questions that focused the third reading and coding of the documents. These addressed the intersection between equity and related concepts that emerged from the analysis, such as accessibility, whether unexpected ideas about equity were put forward, and whether there were programs or standards where equity seemed to be missing. Coding categories were developed, including identifying where equity was explicitly or implicitly mentioned, how equity was measured, concepts like social justice, accessibility and accountability, and specific actions to reduce health inequities. The relationships between coding categories were explored and hypotheses generated to explain their relationship. In the following analysis, we highlight similarities and differences between the Ontario and BC documents in terminology, how equity is conceptualized, what goals are identified, the importance of the social determinants of health (SDOH) and what actions are recommended.

**Table 1:** Questions developed iteratively to analyse key documents

	
***Preliminary questions***	1. Are the following terms mentioned in the documents? Where are they mentioned?
	a. equity, equities
	b. inequity, inequities
	2. Are equity/equities or inequity/inequities explicitly defined in the documents?
	3. In what context is equity/equities or inequity/inequities mentioned?
	4. Is improving equity or reducing inequity an explicit goal?
***Secondary questions***	1. What proxy terms are used to relate to equity?
	2. Is the definition of equity in the ON and BC documents related to social justice?
	3. Is addressing health inequities in the ON and BC documents equated with addressing structural conditions that produce inequities?
	4. Is there reference to the SDOH in the ON and BC documents, and if so, what are the expectations of PH (explicitly or implicitly) in addressing them?
	5. Are there specific examples of certain populations to focus on? E.g. low-income, Aboriginal, etc
	6. To what extent is accountability to address inequities mentioned or considered in the docs?
	7. Are there specific actions recommended or expected from PH in addressing health inequities?
***Tertiary questions***	1. How does equity intersect with accessibility?
	2. Are there unexpected or unusual ideas about equity that appear?
	3. Are there programs or standards where equity seems to have been missed?

## Results

### Ontario

Within the OPHS and its 26 protocols, the terms “equity”, “equities” and “inequity” are seldom mentioned alone, but rather in combination with specific reference to health equity or inequities. The key terms used are “health inequalities”, “health inequities” or “inequities in health”, which are each defined explicitly. In the OPHS, health inequalities are defined as “differences in health status or in the distribution of health determinants between different population groups” [17](p. 4). Some of these are “attributable to the external environment and conditions mainly outside the control of the individuals concerned” and may “lead to inequity”. Health inequities are health inequalities that are deemed “unnecessary and avoidable as well as unjust and unfair” [17] (p. 4). This definition is derived from the World Health Organization [22] and consistent with an influential discussion paper by Whitehead and Dahlgren [23].

The goal of reducing health inequities is very prominent in the Introduction to the OPHS and the Foundational Standard, which underpins all other standards. Achieving health equity is presented as being as important as the improvement of overall population health. The Foundational Standard outlines Board of Health requirements to use data on population health, determinants of health and health inequities to tailor programs to meet local needs, including those of priority populations. There is a suggestion that public health units can achieve both goals of public health simultaneously, i.e. “reduce inequities in health while at the same time maximizing the health gain for the whole population” [17] (p. 13). Reducing health inequities can also be a means of achieving better overall population health with an emphasis on priority populations, defined below. For example, “By tailoring programs and services to meet the needs of priority populations, boards of health contribute to the improvement of overall population health outcomes” and “population health outcomes are often influenced disproportionately by sub-populations who experience inequities in health status” [17](p. 12). Two of the four principles that underpin the OPHS, *need* and *impact*, emphasize the reduction of health inequities. The Population Health Assessment protocol also emphasizes the need to reduce health inequities.

However, this explicit focus on reducing health inequity is not evenly maintained throughout the OPHS. The broad vision of public health presented in the Introduction does not explicitly mention health inequities stating, “the primary focus of public health is the health and well-being of the whole population” [17] (p.2). Few of the specific protocols, which were developed to provide further direction where standardization was identified as needed, actually name reducing inequities as a goal. The strong theoretical commitment to achieving health equity appears to be reconciled with this lack of specificity by identifying the reduction of health inequities as a societal outcome, rather than as a Board of Health outcome. As a societal outcome, it is not solely the responsibility of local public health, but public health is to work with community partners to achieve this goal.

Within the OPHS, the term “priority populations” is often used as a proxy for the need to address health inequities. Priority populations are defined as “those populations that are at risk and for whom public health interventions may be reasonably considered to have a substantial impact at the population level” [17] (p.2). Almost all Protocols refer to priority populations and some explicitly identify certain groups. For example, in the Tuberculosis Prevention and Control Standard, they are identified as “those incarcerated in correctional facilities, Aboriginal peoples and First Nation communities, refugees, recent arrivals to Canada, homeless persons, and those who work closely with these groups” (p. 37). Other ways of implicitly referring to health inequities occur in protocols that call for “all” to have healthy lives, or that label some groups as being “at risk”. For example, the goal of the Child Health Standard is “to enable all children to attain and sustain optimal health and developmental potential” (p. 27). In the Healthy Babies, Healthy Children Protocol, “at risk” is defined as “some risk that a child may not reach his or her full potential”, and “high risk” as “a serious risk that a child may not reach his or her potential”. Addressing the needs of such populations is referred to in the goals of several standards, with the emphasis on the development of individual skills, provision of a safe and supportive environment and influencing the development of health-promoting public policy.

The determinants of health play a prominent role in relation to reducing health inequities in the OPHS. “Addressing determinants of health and reducing health inequities are fundamental to the work of public health in Ontario” [17](p. 2). The determinants of health are also important in identifying priority populations. For example, the Nutritious Food Basket Protocol makes a concrete link, “consider the determinants of health to assist in identifying priority populations and use population health data and information to focus public health action” [17] (p. 1). There is some exploration in the OPHS of why health inequities exist. The Foundational Standard notes, “It is evident that population health outcomes are often influenced disproportionately by sub-populations who experience inequities in health status and comparatively less control over factors and conditions that promote, protect, or sustain their health.” [17](p. 12). These statements highlight the specific need to identify what is unfair and how inequities arise, implicitly acknowledging a commitment to social justice within public health [24].

Three categories of actions to reduce health inequities are discussed in the OPHS. First, there is a focus on surveillance and measurement. For example, the Foundational Standard states, “Population health assessment includes measuring, monitoring, and reporting on the status of a population’s health, including determinants of health and health inequities.” [17] (p. 15). Many protocols discuss the need to identify priority populations through surveillance, but very few discuss taking specific action if health inequities are found. An assumption implicit within the OPHS is that measuring health inequities will lead to action to reduce them.

Second, there is a focus on addressing the accessibility of public health programs and lowering barriers to them. For example, there is a need to “tailor public health programs and services to meet local population health needs, including those of priority populations” (p. 16) and provide “outreach to priority populations to link them to information, programs, and services” [17] (p. 26). For example, products such as vaccines should be distributed in an equitable manner, clinical care should be accessible, and injury prevention and harm reduction services should be targeted to high-risk populations.

Third, the OPHS focuses on partnerships and collaboration to reduce health inequities. “The scope of these standards includes a broad range of population-based activities designed to promote the health of the population as a whole, and with community partners to reduce health inequities” [17] (p. 1). This reflects an understanding that “the ability to influence broader societal changes is the responsibility of many parties.” [17] (p. 13). There are multiple calls for community engagement and to increase the capacity of partners. There is a focus on supporting civil society organizations and engaging them in setting priorities and in implementing programs.

In certain areas of the OPHS, the need to reduce health inequities is absent where it may be anticipated, given what is known about health inequities in Canada [2]. There is little mention of First Nation, Métis and Inuit populations, with the exception of the Tuberculosis Prevention and Control Standard. The Environmental Health and Infectious Disease Program Standards do not discuss health inequities beyond the general concept of certain populations being at greater risk. This is also found in the areas of health promotion and emergency management.

In summary, the OPHS presents a theoretical framework to address health inequities and provides some mechanisms by which local public health can work to reduce them. Most discussion of inequities occurs within the introductory materials. The responsibility of local public health is pragmatically outlined as identifying priority populations, meeting their needs “to the extent possible based on available resources.” [17] (p. 16) and reporting on health inequities to the community, representing one aspect of accountability. Collaboration is seen as central to any effort to reduce inequities. There is a stated need to balance prescriptiveness with autonomy for local public health. Finally, while the OPHS does not delve into why inequities exist, there is a clear recognition of the importance of the SDOH.

### British Columbia

The BC Core Functions Framework and the 15 available MCP papers were included in the analysis and reviewed. Within these documents, a variety of terms are used to discuss health inequities. “Inequalities in health” is the most common, followed by “equitable” and “equity”. The Equity Lens Evidence Review provides the following definition, which is similar to the OPHS, “When inequalities are unfair and avoidable, even unjust, then they become inequities.” [19] (p. i). Related, “vulnerable populations” are defined as “those with a greater-than-average risk of developing health problems… by virtue of their marginalized sociocultural status, their limited access to economic resources, or personal characteristics such as age and gender” [25](p. 3). A number of other terms are used in the BC documents that refer to vulnerable populations. These include “vulnerable groups”, “vulnerable populations of children” and the need to address “health disparities”. Specific groups are named in the Resource Document [19](p. 49) and in several MCP papers (Table 2).

**Table 2:** A comparison of priority groups identified in the BC Core Functions Framework and select Model Core Program Papers

**Framework: Resource document**	**Dental health**	**Healthy infant and child development**	**Healthy living**	**Health emergency management**	**Healthy communities**
Aboriginal people	Adults in care	Teen mothers	Aboriginal people	Residents of group homes	Aboriginal communities
Ethno-cultural communities and people of colour	Low income people	Aboriginal people	People with limited income	Elderly (especially the frail elderly)	Mental health groups
Women, where they are at special risk, or for female-specific conditions	Pregnant women and families	Immigrants, refugees and diverse cultural groups	Immigrant populations	People with physical and mental disabilities	Immigrant groups
Men, where they are at special risk, or for male-specific conditions	People with developmental disabilities		Seniors in care	Ethnic minorities	Low-income seniors
People with disabilities					
Infants and children					
Youth					
Seniors					
People with low incomes					
Residents of remote, rural, or northern communities					
Lesbian, gay, bisexual, and transgendered people					

The goal of reducing health inequalities is prominent throughout. As noted in the Framework, “public health has a duty, as one of its fundamental tasks, to work to reduce inequalities in health” [19] (p. 48). Similar to the OPHS, this task is seen to be as important as improving the overall health of the entire population. This is rooted in the concept of “population health”, which underpins the BC Core Functions Framework [19](p. 8). As noted in the Healthy Living MCP, population health “is an approach to health that aims to improve the health of the entire population and to reduce health inequities among population groups.” [26](p. 3).

An equity lens, along with a population lens, is a cross-cutting feature of the BC Core Functions Framework. The equity lens, which is referenced throughout the documents, is “in place to ensure the health needs of specific populations are addressed” [19](p. 20). The lens is also used for “gathering statistical information from a range of sources on the health status of specific at-risk groups and sub-populations” and to identify “meaningful priorities” [27].

References to the SDOH occur repeatedly throughout the BC Framework and a number of lists of determinants are provided. For example in Health Emergency Management, “disaster vulnerability has been linked to the determinants of health, in particular, income, social status, social supports and personal health” [28](p. 11). This focus on the SDOH is linked to the overarching concept of population health, “which takes into account social, economic, environmental determinants of health, including protective factors, risk factors and vulnerable populations” [25](p. 16). There is an emphasis on the need for each health authority to “assess and report on the determinants” for their population [19] (p. 45). However, there is also an emphasis on taking action. While there is recognition of the limits of the evidence supporting action on SDOH, “this does not diminish the importance of the broader determinants of health and the strategies need (sic) to address them” [19](p. 19).

Actions to reduce health inequities discussed in the BC documents fall into five categories. First, there is a need to quantify health inequities by “gathering statistical information from a range of sources on the health status of specific at-risk groups and sub-populations” [27](p. 18). “The task of public health is to participate in the identification of populations at risk and work to reduce their risk” [19](p. 41). There is also the mention of “developing profiles or snapshots of high-risk populations and sub-groups on a health authority and community level” [26](p. 27).

Second, there is an emphasis on taking action on the SDOH. Examples include addressing housing, community food policies, strengthening community services, local action on urban design and transportation, and bylaws. The Food Security MCP paper is a good example of the idea of taking action to reduce health inequities by focusing on the SDOH. “It is well known that poverty is a major determinant in food insecurity. Significant improvements in food insecurity can be achieved through collaborative efforts to address community food security and poverty issues” [29] (p. 12).

Third, advocacy is identified as key to “addressing fundamental issues such as community poverty, environmental sustainability, social development and economic vitality” [27](p. 4). This is tied to the recognition of the need for “political commitment throughout” and a connection to social justice. In the Resource Docment, [19](p. 9), one figure lists a number of items that relate to social justice under conditions that influence health. These include, “achieving an equitable distribution of income”, “an adequate income for all” and “reduction in the number of families living in poverty”. Further, in the Food Security MCP paper, there is a reference to “fairness and openness” as well as the need for an “equitable distribution of food” [29](p. 26). The Healthy Infant and Child Development MCP is perhaps the most explicit, by calling for public health “to address and work to change the broader community and societal factors that influence child health” [19](p. 26).

Fourth, intersectoral action is seen as key to reducing health inequities, so empowered communities can “take control of the factors that determine their well-being” [27](p. 2). Public health can “build strong social networks and social support” and the idea of collaboration with government, other organizations and with “vulnerable populations”, occurs in several MCP papers. Such collaboration is often for the purpose of affecting policy change. Many papers mention the need to develop partnerships, develop “community coalitions” and take “a leadership role in initiatives that address the determinants of health”.

Fifth, several MCP papers emphasize the need to have general interventions and “targeted interventions” to specific, at-risk or vulnerable groups. The need for access to specific, directed services is noted within Dental Health, “Advocacy for access to dental treatment for vulnerable populations is recognized as a best practice” [30](p. 6). There is also some mention of “equitable access”, “accessible to all” and “improving access/removing barriers” in the Resource Document, but few specifics are given. Providing information to specific groups is noted within the Dental Public Health and the Healthy Infant and Child Development MCP. Related to this, the BC documents have a strong focus on Aboriginal populations, which are mentioned throughout the Framework. They are often cited as a high-risk group or vulnerable population. The Healthy Infant and Child Development MCP specifically lists health inequities between Aboriginal communities and the general population, including higher infant mortality rates, food insecurity and anemia. Similarly, the Unintentional Injury Prevention MCP mentions specific inequities and provides figures. It also mentions the need to use OCAP (ownership, control, access, possession) principles when working with Aboriginal communities [31]. The emphasis is that "Specifically, "it is important that Aboriginal groups have full involvement in the planning and delivery of early childhood health and development programs to families on First Nation reserves as well as Aboriginal families in other communities” [25](p. 14).

There is an emphasis on accountability within the Framework and how it can be achieved. “Consideration may be given, in consultation with the health authorities, to an accountability framework for reducing these inequalities. This may involve reporting on core publicizing health program activities by documenting and making public regional inequalities; by analyzing the factors that contribute to such inequalities; and by reporting on their involvement in advocacy coalitions, agency partnerships, community development, and similar efforts directed at reducing inequalities in access to the basic determinants of health” [19] (p. 49). Similar to the OPHS, there are limitations emphasized as to what public health can do to address health inequities. The determinants of health “may be beyond the control of public health staff or health authorities” [19](p.56). In the Healthy Infant and Child Development MCP, “areas such as income and education levels, housing conditions, and access to child day care programs are outside the authority of the health authority” [25](p. 23). However, this is immediately followed by an emphasis on the advocacy role of public health.

In summary, the importance of addressing health inequities occurs throughout the BC Core Functions Framework and related documents. The use of an equity lens is explicit and has seemingly led to very specific actions outlined in the MCP papers with most naming vulnerable or 'at risk' population. Health inequities between Aboriginal and non-Aboriginal populations are particularly highlighted. There is an emphasis on taking action on the SDOH, particularly through advocacy, which is central to and based on community collaboration.

## Discussion

In this study we have examined the conceptualization and integration of equity within documents that outline standards for local public health in Ontario and BC. These documents were developed as part of the renewal of their respective public health systems.

There are a number of limitations to this study. As a documentary analysis, we are limited in what conclusions we can make about the development of the OPHS and the BC Framework. Without further corroborating evidence, such as that derived from interviews with individuals who participated in public health renewal in each province, it is difficult to know what factors influenced the way that equity has been conceptualized and integrated as described. Such interviews will be undertaken in the next phase of the RePHS study. Also, we cannot be sure how the documents have actually shaped public health practice and this will be important to evaluate over the next several years.

We found a number of similarities between the two sets of documents. For both, reducing health inequities is an important goal and framed as important to improving overall population health. In BC, there is direct naming of health inequities that need to be addressed as well as priority populations that reflect the incorporation of an equity lens. In Ontario, the emphasis is placed on the identification of priority populations and ongoing surveillance and population health assessment. Of note, *First Steps to Health Equity*, a framework developed by two local public health professionals, was made available to coincide with the release of the OPHS [32]. This resource tool supports local public health in meeting the requirements of OPHS to identify, report on and plan to meet the needs of priority populations.

Both the OPHS and the BC Framework identify the limited ability of public health to reduce health inequities if it acts alone. Rather, they emphasize the need for intersectoral collaboration and partnerships through action on the SDOH. This is similar to the steps outlined in a 2004 discussion paper developed by the Federal/Provincial/Territorial Advisory Committee on Population Health and Health Security, which called for leadership and policy development to reduce health disparities, intersectoral collaboration, building community capacity and enhanced knowledge exchange [33].

Differences between Ontario and BC also exist on several levels. A key finding is that the BC process of using an equity lens appears to have translated into more specific and robust methods to address health inequities in the development of BC’s MCP papers. Ontario’s lack of specificity may also reflect the context in which the standards were developed. The Ontario standards are legislated and enforceable and were intended to be resource-neutral. That may have led to greater caution in identifying specific actions around the SDOH that Boards of Health would have been accountable for accomplishing. This caution is perhaps seen also in the language of the OPHS. The term “priority populations” is used instead of more value-laden terms such as vulnerable or marginalized, which are used in the BC documents. The OPHS is also rooted in the history of a previous set of guidelines and could not exceed this mandate, whereas the authors of the BC Core Functions Framework had more freedom to define the scope of their work. For BC, conducting an Evidence Review on equity and incorporating an equity lens seemingly led to being more explicit about the rationale, the specific groups to target and the steps that can be taken by local public health, including advocacy. For Ontario, reducing health inequities was framed as a societal outcome, rather than a Board of Health outcome. Individual public health units should identify local priority populations and develop programs to address their needs. Finally, the BC Framework emphasizes Aboriginal health inequities to a much greater extent than the OPHS. It is not clear whether this reflects the different context in which the standards and framework were developed.

Perhaps not surprisingly [34], an analysis of systemic factors and a deeper questioning about the roots of inequities is not apparent in either set of documents. Whether this should be included in guidance to local public health is a matter of debate [35,36]. In both provinces, identifying priority or vulnerable populations – *a priori* or through viewing epidemiological data through the lens of the SDOH – are a key process to highlight inequities. This raises questions about how most public health practitioners conceptualize differences in health status between groups in their communities. SDOH are not necessarily the same as the structural or systematic factors that lead to inequities [37], and SDOH are not the same as the determinants of inequities [13]. The latter may include social positioning, classism, racism, discrimination and other characteristics of societies that lead to health inequities [38]. As an example, Aboriginal status becomes a proxy for inequity rather than colonization, racism or poverty, which are the root causes of inequities.

It is clear that explicit attention was given to health equity during public health renewal in Ontario and BC. In fact, achieving health equity has been conceptualized as a goal in itself. Progress towards this goal is identified as occurring through action on the SDOH, a paradigm that is consistent with the view that social justice is the foundation of public health [24]. Related to this, there is recognition of the need to move from a focus on health outcomes to broader ideas of societal equity. Manzano and Raphael have framed this as rising levels of discourse around the SDOH [35]. Much work remains to be done. There is little guidance as yet on how to best identify priority populations and how to decide how to balance targeted and universal interventions [12,39].

## Conclusion

This study on public health renewal within Ontario and BC sheds light on an evolving view of health inequities within Canada. Addressing health inequities is a key function of public health and has been embedded within standards used for accountability purposes. Recognizing how equity is conceptualized will assist in developing more explicit, action-oriented and concrete steps for actions. This analysis provides insight into the goals and proposed strategies for achieving health equity and should assist public health professionals at all levels, both in Canada and internationally.

## Appendix: Documents

### 1. Ontario

Ontario Public Health Standards, 2008

Beach Management Protocol, 2008

Children in Need of Treatment (CINOT) Program Protocol, 2008

Drinking Water Protocol, 2008

Exposure of Emergency Service Workers to Infectious Diseases Protocol, 2008

Food Safety Protocol, 2008

Healthy Babies Healthy Children Protocol, 2008

Identification, Investigation and Management of Health Hazards Protocol, 2008

Immunization Management Protocol, 2008

Infection Prevention and Control in Licensed Day Nurseries Protocol, 2008

Infection Prevention and Control in Personal Services Settings Protocol, 2008

Infection Prevention and Control Practices Complaint Protocol, 2008

Infectious Diseases Protocol, 2009

Institutional/Facility Outbreak Prevention and Control Protocol, 2008

Nutritious Food Basket Protocol, 2008

Oral Health Assessment and Surveillance Protocol, 2008

Population Health Assessment and Surveillance Protocol, 2008

Preventive Oral Health Services Protocol, 2008

Protocol for the Monitoring of Community Water Fluoride Levels, 2008

Public Health Emergency Preparedness Protocol, 2008

Rabies Prevention and Control Protocol, 2009

Recreational Water Protocol, 2008

Risk Assessment and Inspection of Facilities Protocol, 2008

Sexual Health and Sexually Transmitted Infections Prevention and Control Protocol, 2008

Tobacco Compliance Protocol, 2008

Tuberculosis Prevention and Control Protocol, 2008

Vaccine Storage and Handling Protocol, 2008

### 2. British Columbia

A Framework for Core Functions in Public Health: Resource Document

Evidence Review: Equity Lens

Model Core Program Paper: Food Security

Model Core Program Paper: Healthy Communities

Model Core Program Paper: Healthy Infant and Child Development

Model Core Program Paper: Healthy Living

Model Core Program Paper: Mental Health

Model Core Program Paper: Reproductive Health and Prevention of Disabilities

Model Core Program Paper: Prevention of Unintentional Injury

Model Core Program Paper: Prevention of Harms Associated with Substances

Model Core Program Paper: Dental Public Health

Model Core Program Paper: Communicable Disease

Model Core Program Paper: Air Quality

Model Core Program Paper: Food Safety

Model Core Program Paper: Water Quality

Model Core Program Paper: Emergency Management

Model Core Program Paper for Prevention of Chronic Diseases

## Abbreviations

BC, British Columbia; MCP, Model core program; MHSPG, Mandatory Health Programs and Services Guidelines; OCAP, Ownership, Control, Access, Possession; OPHS, Ontario Public Health Standards; RePHS, Renewal of Public Health Systems; SDOH, Social determinants of health.

## Competing interests

The authors declare that they have no competing interests.

## Authors’ contributions

ADP assisted with the conceptualization of this study, the development of the methodology, carried out the data collection and analysis, prepared the first draft of the paper and assisted with editing the paper. HM assisted with the conceptualization of this study, the development of the methodology, assisted with data analysis and assisted with developing and editing the paper. BP assisted with the conceptualization of this study, the development of the methodology, assisted with data analysis and assisted with developing and editing the paper. JT assisted with data analysis and with editing the paper. AP assisted with the conceptualization of this study, the development of the methodology, assisted with data analysis and assisted with editing the paper. AC assisted with the development of the methodology and assisted with editing the paper. All authors read and approved the final manuscript.

## References

[B1] Fierlbeck K Public health and collaborative governance Can Public Admin 2010 53 1 1 19 10.1111/j.1754-7121.2010.00110.x

[B2] Butler-Jones D Chief Public Health Officer’s Report on the State of Public Health in Canada 2008 Public Health Agency of Canada, Ottawa

[B3] Naylor CD Learning from SARS: Renewal of public health in Canada 2003 Health Canada, Ottawa

[B4] O'Connor DR Report of the Walkerton Inquiry: The events of May 2000 and related issues 2002 Ontario Ministry of the Attorney General, Toronto

[B5] Whitehead M The concepts and principles of equity and health Int J Health Serv 1992 22 3 429 445 10.2190/986L-LHQ6-2VTE-YRRN 1644507

[B6] Schaefer M Abrantes A Standards for local public health services: where stand the states? Am J Public Health 1985 75 6 649 650 10.2105/AJPH.75.6.649 4003630 PMC1646194

[B7] Weir E d'Entremont N Stalker S Kurji K Robinson V Applying the balanced scorecard to local public health performance measurement: deliberations and decisions BMC Public Health 2009 9 127 10.1186/1471-2458-9-127 19426508 PMC2684743

[B8] Halverson PK Performance measurement and performance standards: old wine in new bottles J Public Health Manag Pract 2000 6 5 vi x 11067655 10.1097/00124784-200006050-00002

[B9] Corso LC Landrum LB Lenaway D Brooks R Halverson PK Building a bridge to accreditation–the role of the National Public Health Performance Standards Program J Public Health Manag Pract 2007 13 4 374 377 17563625 10.1097/01.PHH.0000278030.41573.c2

[B10] Browning P von Cube A Leibrand H Minimum public health standards as a basis for secure public health funding J Public Health Manag Pract 2004 10 1 19 22 15018336 10.1097/00124784-200401000-00004

[B11] Corso LC Lenaway D Beitsch LM Landrum LB Deutsch H The national public health performance standards: driving quality improvement in public health systems J Public Health Manag Pract 2010 16 1 19 23 20009640 10.1097/PHH.0b013e3181c02800

[B12] Commission on the Social Determinants of Health Closing the gap in a generation: health equity through action on the social determinants of health. Final Report of the Commission on Social Determinants of Health World Health Organization. Geneva 2008.

[B13] Graham H Social determinants and their unequal distribution: clarifying policy understandings Milbank Q 2004 82 1 101 124 10.1111/j.0887-378X.2004.00303.x 15016245 PMC2690205

[B14] Signal L Martin J Reid R Carroll C Howden-Chapman P Ormsby VK Richards R Robson B Wall T Tackling health inequalities: moving theory to action Int J Equity Health 2007 6 12 10.1186/1475-9276-6-12 17910778 PMC2100053

[B15] Deber RB Millan K Shapiro H McDougall CW A Cautionary Tale of Downloading Public Health in Ontario: What Does It Say about the Need for National Standards for More Than Doctors and Hospitals? Healthc Policy 2006 2 2 60 75 19305705 PMC2585438

[B16] Ontario Public Health Standards [ https://www.publichealthontario.ca/portal/server.pt?open=512&objID=1191&PageID=0&parentname=CommunityEditor&parentid=12&cached=true&mode=2]

[B17] Ministry of Health and Long-Term Care Ontario Public Health Standards Toronto 2008.

[B18] Ontario Public Health Standards - OPHS presentation template [ http://www.health.gov.on.ca/english/providers/program/pubhealth/oph_standards/ophs/progstds/pdfs/ophs_presentation_template.ppt]

[B19] Population Health and Wellness A framework for core functions in public health: A resource document 2005

[B20] Population Health and Wellness Public Health Renewal in British Columbia: An overview of core functions in public health 2005

[B21] Glaser B Strauss A The discovery of grounded theory: strategies for qualitative research 1967 Aldine, Chicago

[B22] Glossary of terms used - Health Impact Assessment (HIA) [ http://www.who.int/hia/about/glos/en/]

[B23] Whitehead M Dahlgren G Levelling up (part 1): A discussion paper on concepts and principles for tackling social inequities in health 2006 World Health Organization, Copenhagen

[B24] Powers M Faden R Social justice: The moral foundations of public health and health policy 2006 Oxford University Press, Oxford 10.1007/s00103-008-0443-7 18259708

[B25] Model Core Program Paper: Healthy Infant and Child Development [ http://www.phabc.org/pdfcore/Healthy_Infant_and_Child_Development-Model_Core_Program_Paper.pdf]

[B26] Model Core Program Paper: Healthy Living [ http://www.phabc.org/pdfcore/Healthy_Living-Model_Core_Program_Paper.pdf]

[B27] Model Core Program Paper: Healthy Living [ http://www.phabc.org/pdfcore/Healthy_Communities-Model_Core_Program_Paper.pdf]

[B28] Model Core Program Paper: Healthy Emergency Management [ http://www.phabc.org/pdfcore/Health_Emergency_Management_Model_Core_Program_Paper.pdf]

[B29] Model Core Program Paper: Food Security [ http://www.phabc.org/pdfcore/Food_Security_Model_Core_Program_Paper.pdf]

[B30] Model Core Program Paper: Dental Public Health [ http://www.phabc.org/pdfcore/Dental_Health_Model_Core_Program_Paper.pdf]

[B31] Model Core Program Paper: Prevention of Unintentional Injuries [ http://www.phabc.org/pdfcore/Unintentional_Injury_Prevention-Model_Core_Program_Paper.pdf]

[B32] First Steps to Equity: Ideas and Strategies for Health Equity in Ontario 2008-2010 [ http://www.healthnexus.ca/policy/firststeps_healthyequity.pdf]

[B33] Health Disparities Task Group of the Federal, Provincial and Territorial Advisory Committee on Population Health and Health Security Reducing Health Disparities – Roles of the Health Sector: Recommended Policy Directions and Activities Health Canada. Ottawa, 2005.

[B34] Bambra C Fox D Scott-Samuel A Towards a politics of health Health Promot Int 2005 20 2 187 193 10.1093/heapro/dah608 15722364

[B35] Manzano AT Raphael D CPHA and the social determinants of health: an analysis of policy documents and statements and recommendations for future action Can J Public Health 2010 101 5 399 404 21214056 10.1007/BF03404861 PMC6973663

[B36] Kirkpatrick SI McIntyre L The Chief Public Health Officer's report on health inequalities: what are the implications for public health practitioners and researchers? CJPH 2009 100 2 93 95 10.1007/BF03405513 PMC6973718 19839281

[B37] Birn AE Making it politic(al): closing the gap in a generation: health equity through action on the social determinants of health Soc Med 2009 4 3 166 182

[B38] Navarro V What we mean by social determinants of health Int J Health Serv 2009 39 3 423 441 10.2190/HS.39.3.a 19771949

[B39] Kass NE An ethics framework for public health Am J Public Health 2001 91 1776 1782 10.2105/AJPH.91.11.1776 11684600 PMC1446875

